# Fanconi anemia cells with unrepaired DNA damage activate components of the checkpoint recovery process

**DOI:** 10.1186/s12976-015-0011-4

**Published:** 2015-09-18

**Authors:** Alfredo Rodríguez, Leda Torres, Ulises Juárez, David Sosa, Eugenio Azpeitia, Benilde García-de Teresa, Edith Cortés, Rocío Ortíz, Ana M. Salazar, Patricia Ostrosky-Wegman, Luis Mendoza, Sara Frías

**Affiliations:** Laboratorio de Citogenética, Departamento de Investigación en Genética Humana, Instituto Nacional de Pediatría, D.F., México; Programa de Doctorado en Ciencias Biomédicas, Universidad Nacional Autónoma de México, D.F., México; Instituto de Ecología, Universidad Nacional Autónoma de México, D.F., México; C3, Centro de Ciencias de la Complejidad, Universidad Nacional Autónoma de México, D.F., México; Current address: INRIA, Virtual Plants Project Team, UMR AGAP, Montpellier, France; Departamento de Ciencias de la Salud, Universidad Autónoma Metropolitana-Iztapalapa, D.F., México; Instituto de Investigaciones Biomédicas, Universidad Nacional Autónoma de México, D.F., México

**Keywords:** DNA damage, Checkpoint recovery, Boolean network model

## Abstract

**Background:**

The FA/BRCA pathway repairs DNA interstrand crosslinks. Mutations in this pathway cause Fanconi anemia (FA), a chromosome instability syndrome with bone marrow failure and cancer predisposition. Upon DNA damage, normal and FA cells inhibit the cell cycle progression, until the G2/M checkpoint is turned off by the checkpoint recovery, which becomes activated when the DNA damage has been repaired. Interestingly, highly damaged FA cells seem to override the G2/M checkpoint. In this study we explored with a Boolean network model and key experiments whether checkpoint recovery activation occurs in FA cells with extensive unrepaired DNA damage.

**Methods:**

We performed synchronous/asynchronous simulations of the FA/BRCA pathway Boolean network model. FA-A and normal lymphoblastoid cell lines were used to study checkpoint and checkpoint recovery activation after DNA damage induction. The experimental approach included flow cytometry cell cycle analysis, cell division tracking, chromosome aberration analysis and gene expression analysis through qRT-PCR and western blot.

**Results:**

Computational simulations suggested that in FA mutants checkpoint recovery activity inhibits the checkpoint components despite unrepaired DNA damage, a behavior that we did not observed in *wild-type* simulations. This result implies that FA cells would eventually reenter the cell cycle after a DNA damage induced G2/M checkpoint arrest, but before the damage has been fixed. We observed that FA-A cells activate the G2/M checkpoint and arrest in G2 phase, but eventually reach mitosis and divide with unrepaired DNA damage, thus resolving the initial checkpoint arrest. Based on our model result we look for ectopic activity of checkpoint recovery components. We found that checkpoint recovery components, such as PLK1, are expressed to a similar extent as normal undamaged cells do, even though FA-A cells harbor highly damaged DNA.

**Conclusions:**

Our results show that FA cells, despite extensive DNA damage, do not loss the capacity to express the transcriptional and protein components of checkpoint recovery that might eventually allow their division with unrepaired DNA damage. This might allow cell survival but increases the genomic instability inherent to FA individuals and promotes cancer.

## Introduction

The molecular basis of the DNA damage response (DDR) has been largely elucidated through the study of the rare chromosome instability syndromes (CIS) [1] which are cytogenetically characterized by the spontaneous appearance of chromosome aberrations (CA) as well as hypersensitivity to specific DNA damaging agents [2–4]. The best-known CIS include Bloom syndrome (BS) which appears due to mutations in BLM helicase [5, 6] and results in increased sister chromatid exchanges [7], Ataxia Telangiectasia (AT) that shows particular clonal chromosome rearrangements as a consequence of mutations in the checkpoint kinase ATM gene[8–11], and Fanconi anemia (FA) [12] whose phenotype results from mutations in any of the genes that conform the FA/BRCA pathway [13–19] and consists of chromatidic breaks, iso-chromatidic breaks and radial exchange figures among chromosomes. Even if these breaks and radials are predominantly seen in FA, they can also be observed in BS and AT [4, 20]. Although patients affected by CIS display phenotypic similarities, such as growth defects, compromised immunological system and an increased risk to develop cancer [1, 20], each syndrome presents particular phenotypes and pivotal data. Namely, BS shows sun sensitivity [5], AT presents progressive cerebellar ataxia and oculo-cutaneous telangiectases [8], while FA is characterized by congenital malformations and progressive bone marrow failure [21]. The products of these genes interact in the cell’s DNA damage response [1], and thus the deficiency of any of these proteins diminishes the efficiency of a cell to cope with DNA damage, leading to their accumulation.

Given the critical role that these proteins have in the protection of the human genome, certain authors have speculated that survival of CIS patients is an oddity and that cells escaping apoptotic death do so by constitutively inducing alternative replication or DNA damage tolerance pathways, which might contribute to the characteristic mutator phenotypes observed in the CIS [22].

In the particular case of FA, cells are hypersensitive to agents that create DNA interstrand crosslinks (ICL), such as mitomycin C (MMC) or diepoxybutane (DEB) [21]. The treatment of FA cells with MMC or DEB induces a blockage during the G2 phase of the cell cycle and exacerbates the frequency of CAs, including double strand breaks (DSBs) and radial exchange figures [23]. Biallelic mutations in at least one of 18 distinct *FANC* genes can generate FA. The products of these genes interact in the so-called Fanconi Anemia/Breast Cancer (FA/BRCA) pathway [13–18], involved in the repair of the DNA damage generated by intrinsic acetaldehydes and extrinsic ICL inducing agents. Therefore, a deficiency in this pathway results in DNA damage accumulation that might originate congenital malformations, uncontrolled hematopoietic cell death and cancer in FA patients [24–27].

Over the years, the FA diagnosis assays and experimental approaches have shown that a great proportion of FA cells succumb to DNA damage due to their inherent repair deficiencies. However, some cells are able to tolerate high levels of DNA damage and progress into mitosis despite a great amount of CAs. The mechanisms that allow the cells with CAs to omit the DNA damage integrity checkpoints remain uncertain because the more obvious candidate, the G2/M checkpoint, is considered to be properly activated in FA cells [28–30]. Thus, the idea of a malfunctioning checkpoint in FA cells has been ruled out and it is presumed that some other mechanisms are responsible for the checkpoint override in FA cells with unrepaired DSBs.

In recent times, an attenuated G2 checkpoint phenotype, characterized by low levels of CHK1 (NP_001107594.1) and p53 (NP_000537.3), absence of the G2 phase arrest, and arrival to metaphase with a large number of MMC-induced CAs has been described in cells from adult FA individuals [31]. It has been suggested that the G2 checkpoint attenuation could be an important contributor for the increased life expectancy of these FA patients, and that the release of cells with unrepaired DSBs could promote neoplastic transformation [31]. Nevertheless, since non-attenuated FA cells carrying unrepaired DNA damage achieve a correct G2/M checkpoint activation [28–30], the aforementioned mechanism seems to be a particular scenario rather than a general mechanism to enable the resolution of the G2 checkpoint blockage.

Network modeling has been previously used with success to study the dynamics of biological systems [32–37]. Particularly, we developed a Boolean network model (BNM) for the FA/BRCA pathway [38], in which we observed that the inclusion of the checkpoint recovery (CHKREC) node is crucial for the network correct function. In our model, the CHKREC node represents the process that relieves the inhibition of the checkpoint machinery over the mitosis-promoting factor (Cyclin B/CDK1) after a complete DNA damage repair to allow further cell division [39–42]. This node comprises the G2 transcriptional program that activates the expression of genes driving the G2/M transition and the protein program that inactivates the *γ*H2AX histone (NP_002096.1) and checkpoint kinases [43]. We presumed that CHKREC activation might be releasing cells with unrepaired DNA damage in FA mutants. To test this possibility, as well as to validate the inclusion of the node itself in the FA/BRCA network, we used a simplified version of our previously published FA/BRCA pathway BNM and experimentally determined if CHKREC components become activated in FA cells during G2/M during MMC-induced arrest.

## Materials and methods

### Model and simulations

**Fig. 1:**
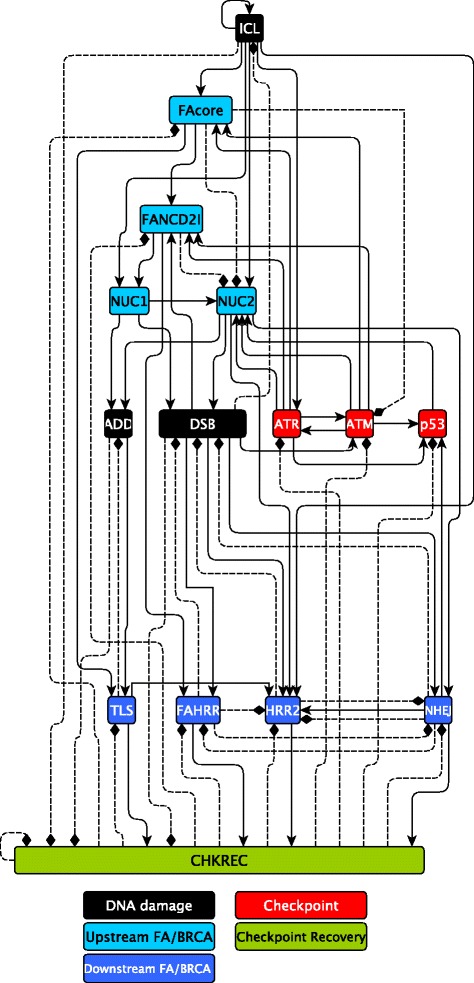
The latest FA/BRCA network. In response to an ICL, the FA/BRCA network responds by blocking the cell cycle through the ATR and ATM checkpoint kinases and their downstream target p53. Similarly, the FA core complex (FAcore) becomes activated and ubiquitinates FANCD2I complex, which in turn recruits DNA endonucleases (NUC1 and NUC2). These endonucleases unhook the ICL generating a DNA adduct (ADD) and a double strand break (DSB). Translesion synythesis (TLS) takes over the ADD while the DSB can be rejoined either by FA/BRCA-dependent Homologous Recombination (FAHRR), FA/BRCA-independent Homologous Recombination (HRR2), or by the error prone Non-Homologous End-Joining (NHEJ) pathways. Finally, we predict that the CHKREC node, composed by the G2/M transcriptional program and checkpoint recovery proteins, turns off the checkpoint and DNA repair proteins. Rectangles represent proteins or protein complexes, pointed arrows are positive regulatory interactions, and dashed lines with blunt arrows are negative regulatory interactions. Readers may refer to [38] for a more detailed description of the FA/BRCA pathway

The simplification of the FA/BRCA network was done by reorganizing the existing 28 nodes and 122 interactions [38], resulting in a deterministic BNM with 15 nodes and 66 interactions (Fig.1 and Table 1) that vastly simplifies the computational analysis while maintaining the qualitative dynamical behavior of the original FA/BRCA network. The simplification was made by collapsing the network components that share functions or belong to a single pathway into one single node. We were careful to preserve all the important functional categories of the network and made sure to recover the behavior of the *wild type* and mutant networks. The modifications and simplification criteria are listed in Table 2.

**Table 1:** Boolean functions for the nodes in the FA/BRCA network

RULES	REFERENCES	
ICL ← ICL ∧¬ DSB	[38]	
FAcore ← ICL ∧ (ATR ∨ ATM) ∧¬ CHKREC	[14, 16, 44–46]	
FANCD2I ← FAcore ∧ ((ATR ∨ ATM) ∨ ((ATR ∨ ATM) ∧ DSB)) ∧	[47–49]	
¬ (CHKREC)		
NUC1 ←ICL ∧ FANCD2I	[50]; [38]	
NUC2 ← (ICL ∧ (ATR ∨ ATM) ∧¬ (FAcore ∧ FANCD2I)) ∨	[51]; [38]	
(ICL ∧ NUC1 ∧ p53 ∧¬(FAcore ∧ FANCD2I))		
ADD ← (NUC1 ∨ NUC2 ∨ (NUC1 ∧ NUC2)) ∧¬ (TLS)	[47, 50, 51]	
DSB ← (NUC1 ∨ NUC2) ∧¬ (NHEJ ∨ FAHRR ∨ HRR2)	[50, 52]	
TLS ← (ADD ∨ (ADD ∧ FAcore)) ∧¬ (CHKREC)	[53, 54]	
FAHRR ← DSB ∧ FANCD2I ∧¬ (NHEJ ∧ CHKREC)	[53, 54]	
HRR2 ← (DSB ∧ NUC2 ∧ NHEJ ∧ ICL ∧¬ (FAHRR ∨ CHKREC)) ∨	[38]	
(DSB ∧ NUC2 ∧ TLS ∧¬ (NHEJ ∨ FAHRR ∨ CHKREC))		
NHEJ ← (DSB ∧ NUC2 ∧¬ (FAHRR ∨ HRR2 ∨ CHKREC))	[49, 52, 55–57]	
ATR ←(ICL ∨ ATM) ∧¬ CHKREC	[58–60]	
ATM ← (ATR ∨ DSB) ∧¬ CHKREC ∨ FAcore	[61, 62]	
P53 ← ((ATR ∨ ATM) ∨ NHEJ) ∧¬ CHKREC	[58, 63, 64]	
CHKREC ← ((TLS ∨ NHEJ ∨ FAHRR ∨ HRR2) ∧¬ DSB) ∨	[52, 53];	
((¬ ADD) ∧ (¬ ICL) ∧ (¬ DSB) ∧¬ (CHKREC))	[38, 65]	

Key references are included. Full discussion of interactions can be found in [38]

**Table 2:** Network model simplification criteria

Node in the	Nodes in the	Simplification criteria	
original BNM	simplified BNM		
ICL	ICL	Unchanged node	
FANCM, FAcore	FAcore	ICL recognition proteins working	
		together in the upstream FA/BRCA	
		pathway	
FANCD2I	FANCD2I	Unchanged node	
MUS81	NUC1	Nuclease mediated	
		ICL incision	
XPF, FAN1	NUC2	Nuclease mediated	
		ICL incision	
ADD	ADD	Unchanged node	
DSB	DSB	Unchanged node	
ATR, CHK1, H2AX	ATR	These proteins act in the	
		Checkpoint pathway	
ATM, CHK2, H2AX	ATM	These proteins act in the	
		Checkpoint pathway	
p53	p53	Unchanged node	
PCNATLS	TLS	This is only a change in name	
FANCJMLH1, MRN, BRCA1,	FAHRR	These proteins act in the	
FANCD1N, RAD51, HRR,		Homologous Recombination	
ssDNARPA		Repair pathway	
——	HRR2	New node representing the	
		alternative Homologous	
		Recombination Repair Pathway	
KU, DNAPK, NHEJ	NHEJ	These proteins act in the	
		Non-Homologous End-Joining	
		DNA repair pathway	
USP1, CHKREC	CHKREC	Global negative regulators	
		of the FA/BRCA pathway	

Simulations were performed for the *wild type* and all possible gain of function or null mutants of the model with synchronous and asynchronous update regimes. Here we report the simulations exploring checkpoint and CHKREC function in *wild type* and FA core mutants. These null mutants were simulated fixing to zero the node of interest.

**Fig. 2:**
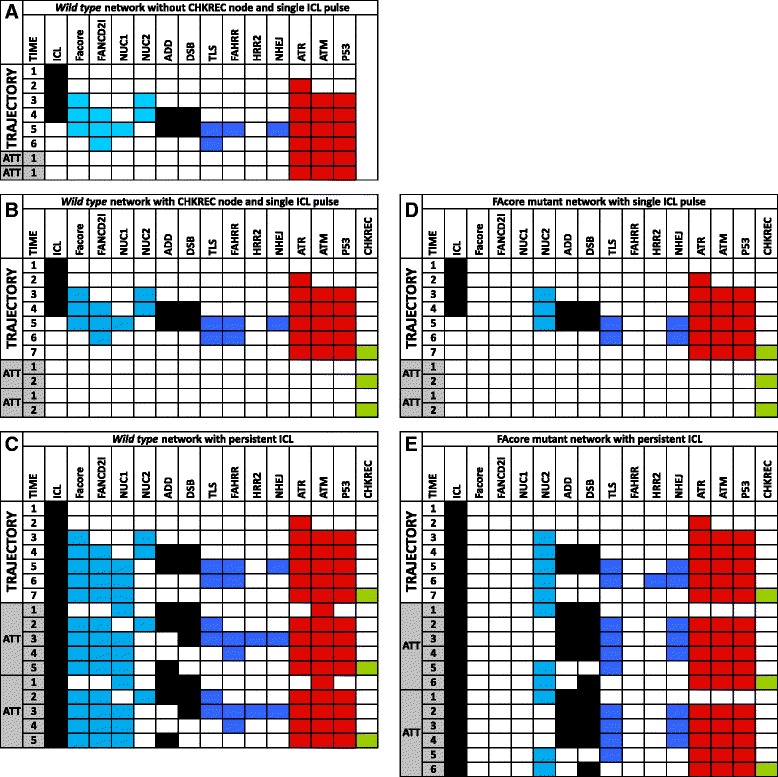
FA network simulations. **a** The current information regarding the FA/BRCA pathway have not uncovered the mechanism that allows the resolution of the G2/M checkpoint after DNA damage and further cell division. **b** Trajectories and attractor of the *wild type* FA/BRCA network under an ICL pulse. In this simulation *wild type* cells repair DNA damage through the FA/BRCA pathway and arrive to CCP attractor after activating the CHKREC node once the damage has been fixed. The inclusion of the CHKREC node, as a checkpoint negative regulator, allows to explore the mechanisms behind cell division after checkpoint resolution. **c** In response to a continuous ICL DNA damage, *wild type* cells arrive to a CCA attractor with activation of the checkpoint and DNA damage repair nodes,the CHKREC node becomes eventually activated in this attractor. **d** Under and ICL pulse FAcore mutant cells activate the NHEJ pathway to repair DNA damage and arrive to a CCP attractor. **e** In response to a continuous ICL DNA damage, FAcore mutant cells concomitantly activate the checkpoint and the CHKREC nodes. Node names are indicated at the topmost row. The leftmost column indicates simulation time steps in arbitrary units. Time steps corresponding to trajectories are indicated and time steps corresponding to attractors are indicated by shaded gray and “ATT”. For illustrative purpose cyclic attractors are represented twice

In these mutants we simulated the response to ICLs, whose presence is dependent on the time that the system requires to turn it off. With our model we simulated two biologically relevant conditions, a short exposure to ICLs, which is supposed to be repaired fast and efficiently by the FA/BRCA pathway (Fig. 2b) and a persistent exposure to damaging agents, which is more difficult to face given the accumulation of damage and saturation of the DNA repair pathway (Fig. 2c). The response to short ICL exposure was simulated in both the *wild type* (Fig. 2b) and FAcore mutant (Fig. 2d) with the ICL value ON only at the starting time step; whereas a continuous exposure to DNA damage was simulated fixing the ICL value to 1 during the entire simulation. We performed exhaustive searches of all possible trajectories and attractors in the system.

### Implementation

The current FA/BRCA network is available through the supplementary file *FAnetwork.r* this file has been tested using R (v3.1.1) package BoolNet (v1.63) [66]. Additionally, the SBML-qual implementation of the model obtained by using the **toSBML()** function of BoolNet is provided as the supplementary file *FAnetwork.sbml*. The generated file was validated using the online service at http://sbml.org/Facilities/Validator/.

### Cell culture and treatments

Lymphoblastoid cell lines from FA-A VU817 (kindly donated by Dr. Hans Joenje, VU University Medical Center) and normal NL-49 (generated in our institution under written informed consent) were maintained in RPMI 1640 medium supplemented with 10 % fetal calf serum, 1 % non-essential aminoacids and 1 % sodium pyruvate (all from GIBCO, Waltham, Massachusetts, USA). During experiments 300,000 cell/ml were exposed to 10 ng/ml of MMC (Sigma-Aldrich Co, St. Louis MO, USA) for 24 h and harvested to evaluate different markers. All the experiments were run by triplicate.

### Chromosome aberration and nuclear division index analysis

For chromosome aberration analysis, colchicine (Sigma-Aldrich Co, St. Louis MO, USA) (final concentration of 0.1 *μ*g/ml) was added to cell cultures one hour before harvesting with the conventional method. Twenty five metaphases per experimental condition were scored by recording the number of chromatid breaks, chromosome breaks and radial figures. A cytokinesis block assay, using 3 *μ*g/ml of cytochalasin B (Sigma-Aldrich Co, St. Louis MO, USA), was implemented to obtain binucleated and tetranucleated cells: after exposing the cells to MMC for 24 h, they were washed, reincubated with fresh cytochalasin B for another 24 h and harvested using a 7:1 methanol:acetic acid fixative. Five hundred cells were scored to quantify the number of micronuclei, mononucleated, binucleated and tetranucleated cells in every experimental condition [67].

### Flow cytometry analysis

To determine cell cycle distribution and mitotic index the cells were fixed with 70 % ice-cold ethanol, washed twice with PBS (GIBCO, Waltham, Massachusetts, USA) and permeabilized with 0.1 % PBS 1X + Triton X100. The MPM2 antibody (CellSignaling, Boston MA, USA) was used to determine the number of cells in M phase. The antibody was marked with the labeling anti-mouse Alexa-Fluor 488 fluorophore from the Zenon Tricolor Mouse IgG Labeling Kit # 1 (Invitrogen, Carlsbad, CA, USA) according to manufacturer instructions. The cells were incubated during 1 h with the antibody, washed with PBS/NGS 10 % and counterstained with propidium iodide (Sigma-Aldrich Co, St. Louis MO, USA). A total of 20,000 events were scored in a FACSCan (Beckton Dickinson, Ontario, CA) cytometer and the analysis was performed using the CellQuest program version 3.2.1.

### RNA extraction and quantitative real-time PCR (qRT-PCR)

Total RNA was obtained employing the combined method of TRIzol (Invitrogen, Carlsbad, CA, USA) followed by RNeasy mini procedure (Qiagen, Valencia, CA, USA), according to manufacturer instructions. Before retro-transcription, 1 *μ*g of total RNA was treated with 0.1 U RNase-free DNase I (Invitrogen, Carlsbad, CA, USA) in 20 mM Tris-HCl, pH 8.3, 50 mM KCl, and 1 mM MgCl_2_ for 15 min at room temperature. The enzyme was inactivated by adding EDTA to a final concentration of 1 mM followed by incubation at 65°C/10 min. Total RNA was retro-transcribed into cDNA using the Transcriptor First Strand cDNA Synthesis Kit (Roche Diagnostics, GmbH, Mannheim, Germany) using anchored-oligo (dT) 18 primer (50-pmol/ *μ*L) and Random hexamer primer (600 pmol/ *μ*L), protector RNase Inhibitor (20 U), and Transcriptor Reverse Transcriptase (10 U). Total RNA and cDNA were quantified using a Nanodrop ND 1000 spectrophotometer (Nanodrop Technologies, Wilmington, DE, USA). Real-time quantitative-PCR (qRT-PCR) was performed by duplicate for each cell line, treatment and biological repeat using 2 *μ*g of cDNA per reaction with the Universal Probes system (Roche Diagnostics, GmbH, Mannheim, Germany) and the Light Cycler Taq Man Master kit (Roche Diagnostics, GmbH, Mannheim, Germany). 7SL (NR_002715.1), *β*2 microglobulin (NM_004048.2) and *β*-actin (NM_001017992.3) gene expression were used as reference. Primers for each gene were designed on-line with the ProbeFinder Software (http://www.universalprobelibrary.com) and manufactured by the Sequencing and Synthesis Unit (IBT, UNAM). The qRT -PCR was carried out in a Light Cycler 2.0 Carousel Roche equipment.

### Protein extraction and immunoblot

Cells were harvested in TLB lysis buffer supplemented with the Complete C protease and phosphatase inhibitors mix (Roche, Mannheim, Germany). Quantification was made with Bradford ready to use reagent (Biorad, Hercules, CA). Total cell protein (10 *μ*g) was separated by 12 % SDS- PAGE, transferred to nitrocellulose membrane (Biorad, Hercules, CA) and incubated with primary antibodies overnight at 4°C followed by incubation with goat-anti-mouse (Invitrogen, Carlsbad, CA, USA) or goat-anti-rabbit (Invitrogen, Carlsbad, CA, USA) HRP tagged secondary antibodies. Bands were visualized with Lumigen on Amersham Hyperfilm (GE Healthcare, Fairfield, CT, USA). Primary antibodies used are listed below: anti-WEE1 (NP_001137448.1) (Abcam, Cambridge, UK), anti-WIP1 (NP_003611.1) (Abcam, Cambridge, UK), anti-pCHK1 Ser345 (Cell Signaling, Boston MA, USA), anti- *γ*H2AX (Genetex, Irvine, CA), anti-p21 (NP_000380.1) (Genetex, Irvine, CA), anti-MYT1 (NP_004526.1) (Genetex, Irvine, CA), anti-Aurora A (NP_003591.2) (Abcam, Cambridge, UK), anti-CDC25B (NP_001274445.1) (Genetex, Irvine, CA) and anti-PLK1 (NP_005021.2) (Abcam, Cambridge, UK); anti-GAPDH (NP_001243728.1) was used as loading control (Genetex, Irvine, CA).

### Statistical analysis

Experimental groups were compared using two way ANOVA, followed by Tukey’s post-hoc test. A difference was considered significant if *p < 0.05.*

## Results

### FA/BRCA network analyses show that CHKREC promotes cell division in FA mutants with DNA damage

Appropriate function of the FA/BRCA pathway guarantees the complete repair of ICLs and correct checkpoint activation impedes cell division upon DNA damage detection [68]. Therefore an accurate model of the FA/BRCA pathway should show cell division after complete DNA damage repair in *wild-type* cells. In our previous work [38], we demonstrated that the inclusion of the CHKREC node is crucial to reproduce correctly the DNA repair behavior. Without CHKREC, as a negative regulator of the checkpoint nodes, the network remains in a permanent arrest after DNA repair (Fig. 2a). Hence, CHKREC provides a mechanism that allows the cell to resolve the checkpoint (Fig. 2b).

We performed synchronous and asynchronous simulations with the updated and simplified version of the FA/BRCA network and observed that the simplified model is able to reproduce all the previously reported results (Fig. 2b and data not shown). Only synchronous simulations are shown given that asynchronous update results in complex trajectories, while preserving the attractors of the original model [38]. Hence, we decided to use our new version of the network model to deeply study the role of CHKREC in the abnormal behavior of FA cells.

Ninety percent of FA patients carry mutations in one of the components of the FA core complex, including FANCA (NP_000126.2), FANCB (NP_001018123.1), FANCC (NP_000127.2), FANCE (NP_068741.1), FANCF (NP_073562.1), FANCG (NP_004620.1), FANCL (NP_001108108.1) and FANCM (NP_001295063.1) [21]. Hence, to study the role of CHKREC in FA cells, we simulated the FA core complex mutant, represented in our model by the FA core node, and compared its dynamic behavior to a *wild type* network.

Our simulations recapitulate two cellular behaviors relevant to DNA damage that are represented by two specific attractors. We denominated them as the cell cycle progression attractor (CCP), and the cell cycle arrest attractor (CCA). The CCP attractor is characterized by the CHKREC-mediated inactivation of every checkpoint node, namely ATM, ATR and p53, followed by CHKREC oscillations. It has been experimentally proven that CHKREC is required for the activation of the genes and proteins that release the G2/M checkpoint to allow cell cycle progression [39, 41–43]. Hence, the cyclic behavior of the CHKREC node in the CCP attractor represents the periodical transition into the cell cycle, and should ideally be reached when DNA damage has been repaired. In our simulations both *wild type* and FA core mutant reach the CCP attractor after an ICL pulse of damage (Fig. 2b,d).

On the other hand, CCA is a cyclic attractor that represents a checkpoint mediated cell cycle arrest that is reached when DNA damage persists and the cell is engaged in a DNA repair process. Once the system has reached CCA there is recurrent activation of the DNA damage repair and the checkpoint nodes, accompanied by CHKREC node activation, thus CHKREC activation might occur during an ongoing CCA but the cell would not divide unless the checkpoint nodes are turned off, which in turn would not occur until the DNA damage has been completely removed. Although more than one combination of node activation patterns can be interpreted as a CCA attractor, all such patterns share the activation of the DNA damage and the checkpoint nodes followed by activation of CHKREC.

In our simulations with a constant ICL damage the *wild type* (Fig. 2c) and FA core mutant (Fig. 2e) networks reach a CCA attractor with checkpoint and CHKREC activation. In the *wild-type* simulation we observe that the checkpoint components are never completely down-regulated in presence of DSBs or during the ICL stimulus, however the FA core mutants have a transient state in which DSBs are activated and the checkpoint components are inactivated, as a response to CHKREC activation in the previous step. This result suggests that FA cells might overcome, through CHKREC activation, the cell cycle arrest despite unrepaired DNA damage.

Our modeling approach has advanced many interesting predictions about the effect of FA mutations during the DNA repair process. In the next section we focused on the one that we considered more general and important. Namely, that CHKREC inhibition over the checkpoint components might allow the division of FA cells even if DNA has not been completely repaired. Hence, we verified if CHKREC activation might occur in FA cells after DNA damage induction allowing their eventual cell division, even in the presence of unrepaired DNA damage.

### CHKREC components are activated in FA cells with unrepaired DNA damage

Cells should divide only after successful and thorough DNA repair [68, 69], which is achieved through efficient DNA repair and accurate G2/M checkpoint activation. FA core mutants are DNA repair deficient but G2/M checkpoint proficient, therefore the fact that they are able to divide despite a strong G2/M checkpoint activation and carrying unrepaired DNA damage is remarkable. Our BNM anticipates that turning off a DNA damage-induced G2/M checkpoint might occur through CHKREC activation, thus allowing cell division. We verified this prediction by following the transit through G2 and M phases in the presence of DNA damage in *wild type* (NL49) and FA-A (VU817) cell lines exposed to MMC.

**Fig. 3:**
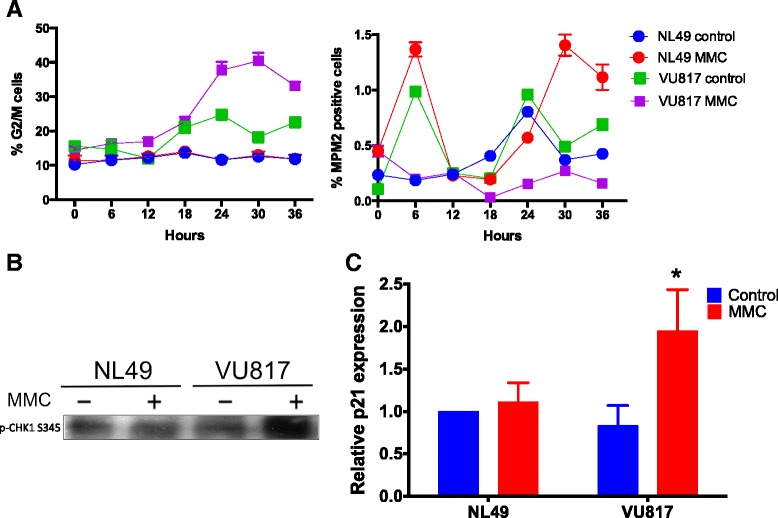
FA cells arrest at G2 phase in response to MMC. **a** Flow cytometry analysis showing accumulation of FA cells in G2 in response to MMC (left panel) and diminished number of FA mitotic cells compared to normal cells (right panel). **b** FA cells activate CHK1 kinase in response to MMC treatment. **c** FA cells increase the expression of p21 mRNA as showed by qRT-PCR analysis (*n* = 3 independent experiments, *p < 0.05*)

First, we evaluated checkpoint activation using several markers. Using PI cell cycle flow cytometry analysis we observed that treatment with MMC induces an over time increase in the number of FA-A cells arrested in G2 when compared to normal cells (Fig. 3a, left panel), as well as a reduction in the number of mitotic cells (MPM2+ cells in Fig. 3a, right panel), accompanied by a lag of approximately 6–12 hours in the peak of MPM2+ cells in both MMC-treated FA and normal cells, compared to their respective untreated controls (see 24 and 30 hrs of MMC treatment). However normal MMC treated cells have the highest peak as they deliver a bigger number of cells into M phase. This lag might indicate that, while repairing the MMC-induced DNA damage, the cells postpone the resolution of the G2 checkpoint. This arrest is shorter in normal cells given that they repair in a more efficient way thus having a more prominent contribution to the mitotic index when compared to FA cells. The highest percentage of MPM2+ cells of MMC treated normal cells might indicate a sharp delivery of previously G2 arrested cells, contrary to a smooth delivery of untreated normal cells.

In FA-A cells treated with MMC we also observed increased CHK1 phosphorylation (Fig. 3b), a classical checkpoint activation marker, along with increased p21 mRNA expression (Fig. 3c). p21 is the main p53 target and is an important player for cell cycle arrest. The expression of this gene shows that the cell is committed to cell cycle arrest and its continuous expression is necessary to prevent cell division in cells that carry unrepaired chromosomes [68, 69]. These experiments show that FA-A cells are able to activate mechanisms that halt cell cycle progression at the G2 phase upon DNA damage induction.

We then evaluated if FA-A and normal cells were able to divide despite unrepaired DNA damage. We quantified the cell division capacity after MMC treatment by performing a cytokinesis block assay with cytochalasin B (CB). Meanwhile, the DNA damage was evaluated by recording the frequency of micronuclei in multinucleated cells and the frequency of CAs in metaphase spreads.

**Fig. 4:**
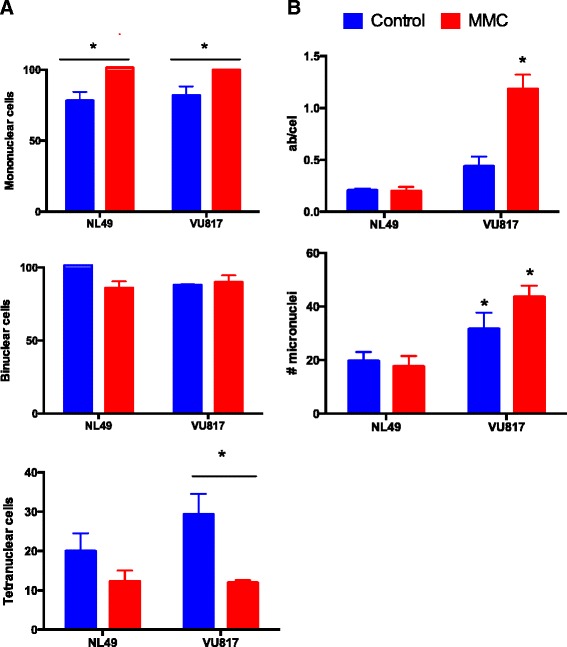
FA cells divide despite MMC treatment and cell cycle arrest. **a** The number of mononucleated cells (upper panel), binucleated cells (middle panel) and tetranucleated cells (bottom panel) was quantified after exposure to MMC (24 h) and Citochalasin B (48 h). The number of basal binucleated and tetranucleated cells was counted without Citochalasin B treatment and rested from the total number (data not shown). **b** Despite G2 arrest FA cells arrive to mitosis and divide with unrepaired DNA damage as demonstrated by two independent DNA damage analyses, increased chromosome aberrations (upper panel) and increased micronuclei in cells that have reached one division (bottom panel) (*n* = 3 independent experiments, *p < 0.05*)

CB experiments showed that treatment with MMC increased the proportion of mononucleated cells (cells that still do not divide due to G2 halt) (Fig. 4a upper panel), while the number of binucleated cells irrespective of the cell type (NL49 or VU817) or the addition or not of MMC remained the same (Fig. 4a middle panel). Remarkably, MMC treatment reduced significantly the number of tetranucleated FA cells (Fig. 4a bottom panel). On the other hand, the analysis of metaphase spreads showed that FA-A cells reached mitosis with a significantly higher frequency of CAs (Fig. 4b upper panel) than normal cells, and were able to divide despite unrepaired DNA damage, i.e. micronuclei (Fig. 4b bottom panel). These experiments show that FA-A cells first arrest in response to DNA damage but eventually reach cell division regardless of CA.

**Fig. 5:**
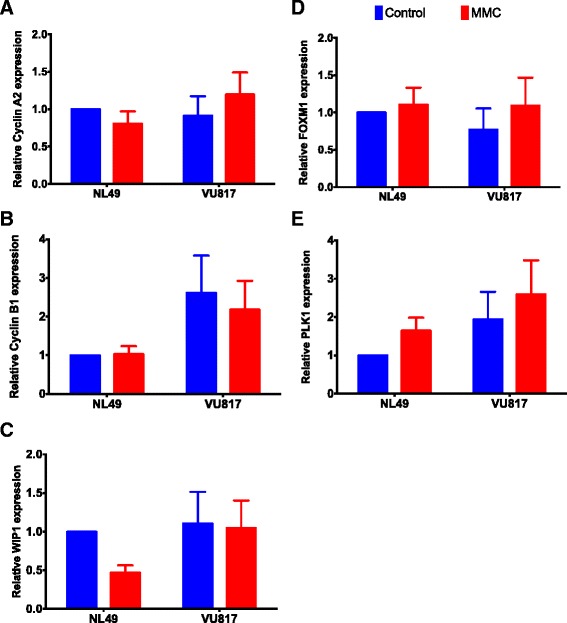
FA cells have a gene expression pattern compatible with checkpoint resolution despite DNA damage. Gene expression analysis of the genes belonging to the G2 transcriptional program did not show differences in the expression of these genes despite MMC treatment. Cyclin A2 (**a**), Cyclin B1 (**b**), WIP1 (**c**), FOXM1 (**d**) and PLK1 (**e**) (*n* = 3 independent experiments). No statistically significant differences were found in the gene expression patterns among groups

As suggested by our model, CHKREC activation could be relieving cell cycle arrest mediators, leading the cell to divide. To determine if the CHKREC components became active in MMC treated FA cells, thus allowing their eventual division, we evaluated some molecular markers relevant for CHKREC and cell division. We analyzed by qRT-PCR the expression of the G2 transcriptional program, whose protein products are necessary for the G2/M transition; namely, Cyclin A2 (*CCNA2*, NM_001237.3), Cyclin B1 (*CCNB1*, NM_031966.3), WIP1 (*PPM1D*, NM_003620.3), *FOXM1* (NM_001243088.1) and *PLK1* (NM_005030.4) [16, 27, 70]. Our results show that the expression levels of these genes remain unaffected in FA-A cells, compared to wild type cells (Fig. 5a–e). Importantly, these genes are expressed in a cell cycle-dependent manner and are necessary for G2 phase completion [43], thus if they remain unchanged after MMC treatment, suggests that this program remains poised for resolution of the G2 cell cycle blockage in FA cells, even with incomplete DNA repair.

Our BNM and these experimental results indicate that FA-A cells are able to block the cell cycle progression in G2 but eventually recover. Simulations with the BNM showed also that co-activation of G2 checkpoint and CHKREC components might occur in arrested FA cells, therefore, additional to CHK1, we evaluated other protein markers to asses key G2 checkpoint and CHKREC activation 24 h after MMC treatment. The G2 checkpoint markers included: WEE1, MYT1, p21 and *γ*H2AX; while CHKREC activation markers consisted of: WIP1, Aurora A, PLK1 and CDC25B.

**Fig. 6:**
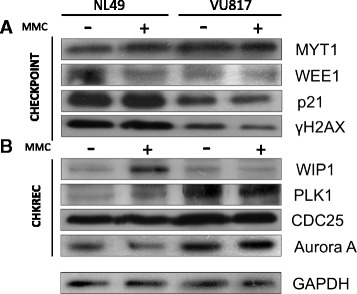
FA and normal cells co-express checkpoint and CHKREC proteins. **a** Western Blot analysis of checkpoint proteins. **b** Western blot analysis of CHKREC proteins. FA cells increase the amount of some G2 blockage proteins, but have a reduction in others. Although CHK1 (Fig. 4 b) and MYT1 show increased signal, WEE1, *γ*H2AX and p21 protein appear as diminished in FA cells, this weakens the checkpoint blockage, which is eventually overwhelmed by CHKREC signaling (*n* = 3 independent experiments, see also Fig.7)

**Fig. 7:**
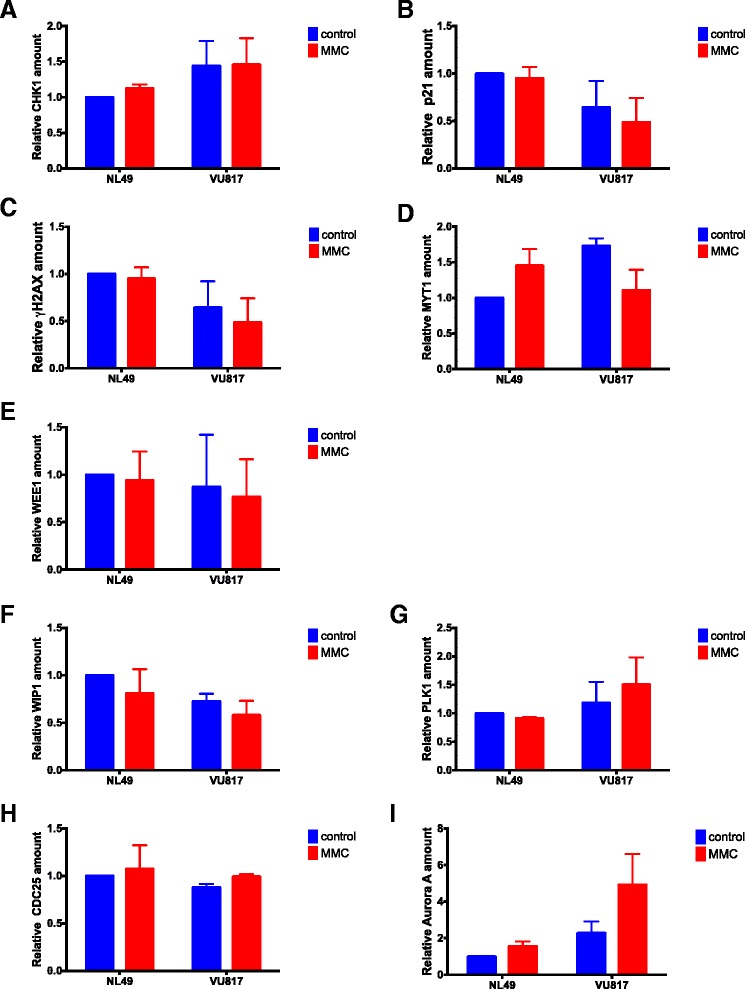
Western Blot densitometry analysis. Checkpoint proteins (**a**–**e**) and CHKREC proteins (**f**–**i**). (*n* = 3 independent experiments). No statistically significant differences were found in the protein expression patterns among groups

We observed that CHK1 activity is increased after MMC treatment in FA-A cells (See Fig. 3b), but other checkpoint components, namely WEE1, p21 and *γ*H2AX (Fig. 6a), have reduced levels. Remarkably, we also observed concomitant activation of CHKREC proteins PLK1, CDC25B and Aurora A (Fig. 6b) in damaged FA cells. Thus indicating that despite a strong CHK1 signal that leads to cell cycle progression blockage, FA-A cells co-express the components that might dampen the DNA damage signaling and allow an eventual CHKREC despite an elevated amount of CA. In agreement, we observed in FA-A cells reduced levels of the histone *γ*H2AX, a DNA damage signaler and WIP1 phosphatase target. Notably, we also observed weakening of p21 protein signaling, which has also been correlated with CHKREC activation [71]. These results suggest that signaling of DSBs might be weakened at this time-point, triggering full CHKREC activation and cell division despite a strong CHK1 signaling and high levels of CAs (relative protein amount for all the markers can be seen in Fig. 7).

These experimental results show that the CHKREC is being activated in FA cells carrying CA to a similar extent as normal undamaged cells, this CHKREC induction might allow the escape of G2 with unrepaired DNA damage and cell division. The correlation between the nodes of the network and the experiments performed can be seen in Table 3.

**Table 3:** Correlation between experimental validations and nodes in the FA/BRCA BNM

Process	Nodes in the	Experimental markers	Validated role	References
	FA/BRCA BNM	used in this study	in the BNM	
DNA damage	ICL	MMC	FA cells are	[21, 25, 26]
induction			hypersensitive	
			to ICL inducing	
			agents	
Upstream	FAcore	Non-evaluated	ICL recognition	[14, 16, 17]
FA/BRCA	FANCD2I		proteins	
pathway	NUC1			
	NUC2			
DNA repair	ADD	*γ*H2AX,	ICLs are unhooked	[13, 21]
intermediaries	DSB	CA in metaphase	by FA core-recruited	
		spreads	DNA-endonucleases	
			that generate a DSB	
			and an ADD	
Downstream	TLS	Non-evaluated	The ADD and DSB	[14, 15, 18, 54]
FA/BRCA	FAHRR		are repaired by TLS	
pathway			and FA-dependent	
			downstream homologous	
			recombination repair,	
			respectively. FA cells	
			accumulate DSBs	
Alternative	HRR2	Non-evaluated	FA cells use	[49, 56]
DNA repair	NHEJ		alternative DNA	
pathways			repair pathways,	
			mainly NHEJ	
			HRR2 is a	
			criptic repair choice	
Checkpoint	ATR	Cell cycle arrest	Upon DNA damage	[27, 28, 31]
	ATM	in G2, pCHK1-S341,	normal and FA	
	p53	p21 gene expression,	cells activate	
		MYT1, WEE1, p21	the G2/M checkpoint	
		proteins		
Checkpoint	CHKREC	MPM2 mitotic index,	The checkpoint	[83, 84] and this work
recovery		cytokinesis block assay,	is inactivated by	
		G2/M transcriptional	CHKREC after	
		program, WIP1, PLK1,	DNA repair	
		CDC25, Aurora A	FA cells seem to have	
		proteins	a lower threshold for	
			CHKREC activation	
			compared to normal cells	

## Discussion

Several methods are used to model and analyze biological systems [72–74]. These methods analyze the topology of the network or the kinetics of the system specifying the flux of information through a continuous model or a logical model [74].

Continuous models represent the temporal dynamics of biochemical processes with considerable detail, but are highly dependent on the values of free parameters (initial protein concentrations and rate constants), whose estimation might be challenging as networks get larger [73, 75]. Logical models rely on qualitative knowledge [72]. Logical BNM are the minimal computational model necessary to obtain a meaningful idea about the dynamics of a regulatory network and are useful when detailed enzymatic information is missing [75, 76]. Many molecular regulatory systems show binary behaviors or act like bistable switches [77], thus the binary or discrete representation of BNM can adjusts to them and predict sequence patterns of proteins and gene activities with less parameters than a continuous model. Although BNM have been used for modeling several systems [32–37], they might not be appropriate if the system has continuous values or if knowledge on the network architecture is lacking [75].

We have developed a binary BNM that recapitulates in a simple manner the response to ICLs mediated by the FA/BRCA pathway [78]. Given that the different components of the network might remain unchanged, up-regulated or down-regulated instead of binary, an additional representation of the FA/BRCA network as a discrete ternary logical network might be also feasible [79], however a binary BNM resulted optimal given that our system presents gene expression showing a pattern of binary states (over-expressed, under-expressed) or protein concentrations that can reach a saturation regime (full activation) or remain in small concentrations (inactive). In addition the change to a ternary system would increase the possible states of the system from 32,768 to 14,348,907 states, thus augmenting the computational work.

Our modeling of the FA/BRCA regulatory network has led to the observation that CHKREC is a mechanism conferring stability to this system in *wild type* and FA cells ([38] and this work). CHKREC is fully activated once the G2/M checkpoint has been satisfied leading to the division of the cell [42]. CHKREC is mainly composed of phosphatases, such as WIP1, that inactivate the G2 checkpoint and protein-kinases that release the cell cycle blockage, such as Aurora A and PLK1 [41, 80]. Notably, the negative circuits mediated by CHKREC seem to be a central part of the control system of the FA/BRCA network: they are activated when the system induces the expression of its own inhibitors, and are necessary to attenuate the stimulatory signals arising from DNA damage (Fig. 1 and Fig. 2).

When simulating mutants, we noticed that CHKREC function inactivates the checkpoint in FA core mutants despite unrepaired DNA damage, thus resolving the G2/M checkpoint arrest and allowing cell division. Therefore we should notice elevated/unchanged levels in the expression, quantity or activity of CHKREC components in FA cells with damaged DNA compared to undamaged normal cells, indicating that FA cells conserve checkpoint resolution capacity and are poised for cell division when the DNA damage checkpoint response ceases. To test the function of the CHKREC node, we experimentally evaluated the cell division capacity as well as checkpoint/CHKREC activation in FA-A lymphoblasts after induction of DNA damage with MMC.

We evaluated the G2 blockage and found accumulation of FA-A cells into the G2 phase compartment after induction of DNA damage (Fig. 3a left panel) and a reduced number of FA-A mitotic cells in comparison to normal cells after MMC exposition (Fig. 3a right panel). We also detected high CHK1 phosphorylation levels (Fig. 3b) as well as high p21 gene expression (Fig. 3c) in FA-A cells. CHK1 is a key protein kinase that transduces the DNA damage signaling, and p21 is a direct p53 transcription target, therefore an increase in p21 activation is the result of p53-increased activity, thus demonstrating that FA cells achieve a correct activation of the checkpoint that blocks the G2/M transition [27, 28]. p21 is a negative regulator of Cyclin B/CDK1 complex and is necessary to avoid the G2/M transition in presence of DNA damage [81]. Thus, CHK1 phosphorylation and p21 expression augment when a cell is exposed to DNA damaging agents and would be expected to drop-off once a cell has repaired the DNA damage [82].

When we evaluated the cell division capacity in a CB block assay, we did not observe differences in the frequency of binucleated cells between normal and FA-A cells (Fig. 4 middle panel), although MMC limited tetranucleated cells production in both cell types (Fig. 4c bottom panel). These results show that, under these experimental conditions, both FA-A and normal cells divide to a similar extent after induction of DNA damage by MMC.

The capability of FA cells to divide with unrepaired DNA damage was evaluated by quantifying the frequency of DNA damage induced by MMC in cells committed to divide by scoring CAs in metaphase spreads, as well as in cells that have already performed cell division by quantifying the micronuclei observed in binucleated and tetranucleated cells. Our results showed, in both assays, that FA-A cells exposed to MMC carry significant DNA damage during mitosis and, nonetheless divide (Fig. 4b).

**Fig. 8:**
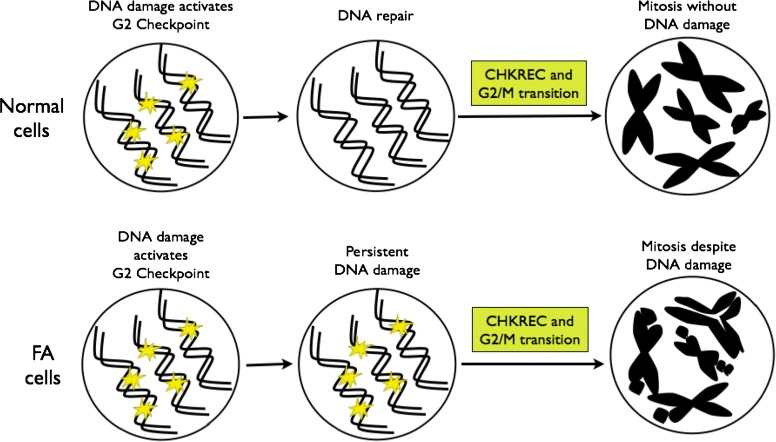
CHKREC components activation in normal and FA cells. After DNA damage induction, the cell activates the DNA damage integrity checkpoints, the G2/M checkpoint specifically avoids the transition of the cell from G2 to M phase with unrepaired DNA damage. Once the DNA has been repaired, the G2/M checkpoint is satisfied and the cell activates the CHKREC, a process that inactivates checkpoint proteins and promotes cell division. Upper panel. Normal cells activate CHKREC with repaired chromosomes. Bottom panel. FA cells activate CHKREC despite unrepaired chromosomes. The specific mechanism triggering this inappropriate CHKREC activation in FA cells remains unknown

Our BNM allowed us to propose that CHKREC function in FA cells might ignore in a certain level the presence of unrepaired DNA damage and could be responsible for their division, therefore we expected that normal and FA-A cells would have similar activation levels of the CHKREC components. To test this possibility, we measured the expression of the G2 transcriptional program genes that promote CDK activity and progression into mitosis, namely *WIP1, Cyclin A2, Cyclin B1, PLK1, CDC25* and *FOXM1* [43] (Fig. 5a–e) and evaluated the activation of some of the proteins involved in checkpoint and CHKREC (Fig. 6). In the first assay we observed that the expression of the genes that enable CHKREC and cell division are similar in normal/undamaged cells and damaged FA cells, even when FA cells carry a higher number of CAs. In addition, we observed co-expression of checkpoint and CHKREC proteins in FA cells treated with MMC (Fig. 6 and 7), indicating that damaged FA cells are poised for an eventual cell division despite DNA damage (Fig. 8).

Checkpoint activation, cell cycle arrest and DNA repair require a great number of protein posttranslational modifications for their establishment. Dedicated enzymes that remove these modifications or degrade modified proteins allow checkpoint silencing and recovery [84]. WIP1 phosphatase and PLK1 kinase emerge as the coordinators of checkpoint silencing and recovery, respectively, however if there exist a certain order in their activation remains elusive. In general terms for cell division, Cyclin B levels must gradually increase, while CDC25 phosphatase should remove any inhibitory phosphorylation of CDK1, thus promoting Cyclin B/CDK1 complex formation and mitotic entry. However, after induction of DNA damage, the G2 checkpoint inhibits CDK1 activity through p21, whilst WEE1 and MYT1 kinases degrade CDC25, avoiding mitotic entry [42, 70, 80].

WIP1 phosphatase dephosphorylates ATM, p53, CHK1, CHK2, *γ*H2AX and the p(S/T)Q motif originally modified by ATM and ATR [85, 86]. During CHKREC, Aurora-A kinase activates PLK1, which in turn targets WEE1 for proteasomal degradation and releases CDK1 from blockage [42, 87–89], in addition PLK1 interferes with CHK1, CHK2 and p53 stability, thus it also has an active role turning-off the DNA damage checkpoint [40].

In Fig. 6b we observe that the concentration of WIP1 is increased in normal cells and reduced in FA cells; on the contrary PLK1 is reduced in normal cell and increased in FA cells. Interestingly, PLK1 activity is redundant in unperturbed mitotic entry whereas it becomes essential in CHKREC after DNA damage [88, 89], consistently it is activated in our experiments in damaged FA-A cells. As FA cells carry spontaneous unrepaired DNA damage, this implies that their transition through G2 is always perturbed to a certain extent, thus PLK1 should become essential for FA cells survival. Given this, PLK1 over-activation must be involved in the adaptation of FA cells to DNA damage. Recent evidence shows that PLK1 activity is gradually increased during an ongoing DNA damage-induced cell cycle arrest and if the activity of the kinase exceeds beyond a certain level, the cell progresses to mitosis despite DNA damage persistance [83]. G2 checkpoint recovery might thus represent a checkpoint adaptation, where DNA damage triggers an arrest whose duration is not necessarily conditioned by DNA repair [84]. Regarding this, PLK1 might have a more critical role than WIP1 in the delivery of FA cells with unrepaired DNA damage from the G2 arrest, or WIP1 is acting before than the time-point that we are evaluating in this assays, hence we are not able to detect WIP1 protein (Fig. 6b). The distinction between both possible scenarios deserves further research.

A final aspect to be considered are the findings of Ceccaldi and coworkers [31], who described an attenuated G2/M checkpoint activity in adult FA individuals that, concomitantly to low CHK1 and p53 protein levels, allowed the escape of unrepaired DNA damage. Although they demonstrate that downregulation of the ATR-CHK1 axis is responsible for this phenotype, it remains elusive if this reduced checkpoint activity might be due to CHKREC over-activation or ectopic activity. In this study we set the basis to explore this possibility in FA individuals with an attenuated G2/M checkpoint and the general mechanism allowing G2/M resolution in non-attenuated FA individuals. Further, modeling the full interaction between the G2/M checkpoint and CHKREC, as well as a systematic inhibition of CHKREC components in FA cells, will shed light into the intricate interactions between these two processes.

Our results show that highly damaged FA-A cells preserve the capacity to divide after a cell cycle arrest induced by DNA damage, a result that is consistent with our BNM FA core null mutant simulations. Nonetheless, the definition of the specific trigger for cell division remains unknown. To our judgment, the CHKREC hypothesis became the most relevant hypothesis emerging from our BNM given that CHKREC promotion might enable cell survival and amelioration of blood cell counts in pancytopenic FA patients, however CHKREC overexpression might also lead to exhaustion of the hematopoietic stem cell compartment as well as selection of malignant clones. Therefore, the thorough study of this process becomes relevant for the understanding of hematopoiesis and carcinogenesis in a FA background.

## Conclusion

In this study we propose through network modeling that CHKREC, a program necessary for cell division after DNA damage, becomes activated in FA core mutant cells with unrepaired DNA damage. We experimentally show that highly damaged FA-A cells have CHKREC expression levels similar to those observed in normal undamaged cells, thus FA-A cells might ignore the presence of broken chromosomes through this process. We observed that despite a prominent G2 arrest after MMC exposure, FA cells were able to activate the mechanisms that allow cell division (Fig. 8).

FA cells are prone to apoptosis due to their DNA repair defects, however a great quantity of them divide in spite of unrepaired DNA damage, thus allowing the survival of FA individuals. The study of the mechanisms that allow FA cells to survive may help to develop novel therapies designed to promote hematopoiesis, as well as to avoid the division of malignant clones in FA patients.
